# Frailty assessment and outcomes in primary care for patients with diabetes during Ramadan: implications for risk evaluation and care plans

**DOI:** 10.3389/fmed.2024.1426140

**Published:** 2024-09-30

**Authors:** Latifa Mohammad Baynouna Alketbi, Bachar Afandi, Nico Nagelkerke, Hanan Abdubaqi, Ruqaya Abdulla Al Nuaimi, Mariam Rashed Al Saedi, Fatima Ibrahim Al Blooshi, Noura Salem Al Blooshi, Aysha Mohammed Al Aryani, Nouf Mohammed Al Marzooqi, Amal Abdullah Al Khouri, Shamsa Ahmed Al Mansoori, Mohammad Hassanein

**Affiliations:** ^1^Ambulatory Healthcare Services, Abu Dhabi, United Arab Emirates; ^2^Tawam Hospital, Abu Dhabi, United Arab Emirates; ^3^College of Medicine and Health Sciences, United Arab Emirates University, Abu Dhabi, United Arab Emirates; ^4^Dubai Hospital, Dubai, United Arab Emirates

**Keywords:** frailty, diabetes mellitus, fasting, adverse events, risk assessment

## Abstract

**Background:**

Frailty is a critical concern for older adults, impacting their susceptibility to adverse events and overall quality of life. This study aimed to determine the frailty status of patients 60 years or older in Abu Dhabi Ambulatory Healthcare Services (AHS) and assess its relation to the stress exerted by Ramadan fasting and the occurrence of any adverse outcomes.

**Methods:**

In this prospective observational study, participants were included if the attending physicians used the IDF-DAR risk stratification assessment tool. A tele-interview was conducted to complete the FRAIL score within 6 weeks before Ramadan 1,444 (CE 2022). The outcome was assessed through another tele-interview and an electronic medical record review after Ramadan.

**Results:**

According to the FRAIL assessment tool, among the 204 patients aged 60 years or older included in the study, 109 (53.4%) were classified as either frail or pre-frail. In total, 20 (9.8%) patients were frail, that is, 1 out of 10, and 89 (43.6%) were pre-frail. The remaining 95 (46.6%) patients were robust. Using logistic regression to assess the occurrence of adverse outcomes after Ramadan fasting, a higher frailty score was identified as the third independent risk factor [B = 0.4, OR = 1.5 (1–2.02–1.86), and *p* = 0.039] for experiencing an adverse event. The identified factors associated with frailty were age, increased albumin-to-creatinine ratio (ACR), chronic kidney diseases (CKDs), and ischemic heart diseases (beta = 0.27, *p* = 0.003; beta = 0.24, *p* = 0.004; beta = 0.2, *p* = 0.039; and beta = 0.18, *p* = 0.041, respectively). One-third of the frail patients had an event, while the incidence in pre-frail patients was 11.2%, and among the robust patients, the incidence was 6.3%. Physicians’ global assessment of frailty did not align well with the structured FRAIL scoring. Only five (25%) out of the 20 patients identified as frail by the FRAIL assessment tool were also judged as frail or having cognitive function impairment by the physicians’ global assessment tool.

**Conclusion:**

Frailty is prevalent among elderly patients with diabetes. Disparity exists between subjective and objective frailty assessments, emphasizing the need for standardized evaluation methods. Using the FRAIL tool is recommended for patients aged 60 or older with diabetes in Abu Dhabi.

## Background

Frailty, a term often used to describe a group of older adults with compromised health, lacks a specific diagnostic definition, and a consensus on its precise characterization remains elusive (1). Nevertheless, the vulnerability of frail individuals to adverse events and their diminished quality of life have driven a growing body of research in this field (2). Cross-sectional studies have indicated that frailty affects approximately 7% of individuals over the age of 65, with its prevalence increasing in tandem with age and surging to potentially exceed 45% among those aged 85 and older (3). Notably, baseline frailty and the annual rate of change in frailty exhibit relatively independent associations with the risk of mortality. Each one-unit increase in the annual frailty change amplifies the risk of death by more than 5-fold (3).

The diverse landscape of frailty assessment tools and scores mirrors the non-linear progression of frailty with age. These tools uniformly show that mortality risk escalates with increasing frailty scores, with a risk increase ranging between 1 and 3.6% per year on a logarithmic scale. Furthermore, frailty scores across different scales demonstrate dose–response relationships with 5-year mortality (4). Contemporary patient care has witnessed a surge in the application of frailty assessment tools to aid in decision-making and the development of patient care plans. The primary objective is to detect high-risk patients early and manage their frailty to mitigate adverse outcomes. However, it is essential to recognize that aging populations exhibit significant heterogeneity in health status (4). Therefore, further research in this realm is warranted, with the potential to unveil new therapeutic approaches. These approaches might include delving into the mechanisms underlying frailty and its influence on disease expression, offering the promise of novel interventions to ameliorate frailty (2).

The majority of frailty studies have traditionally been conducted in North America. However, with the global expansion of the geriatric population, there is a growing need for research on the validity of frailty assessment tools and the prevalence of frailty on an international scale. This is particularly crucial given that a single study has underscored the existence of substantial differences among these assessment scales in terms of content validity, feasibility, and their capacity to predict all-cause mortality. It is evident that these frailty scales capture related yet distinct groups, and there is a significant potential for improvement by weighting items in these scales. However, this improvement must be balanced with considerations of specificity, predictive power, and generalizability, necessitating further evaluation (5).

The present study serves as an extension of an earlier investigation aimed at validating the IDF-DAR risk stratification assessment tool for patients with diabetes fasting during Ramadan (6). Within this broader context, the FRAIL assessment tool was incorporated into the evaluation of the study subjects. It includes five questions focusing on comorbidities, weight loss, and exercise tolerability, such as walking and stair climbing. This study is dedicated to reporting the prevalence of frailty among patients with diabetes who are 60 years or older, in addition to identifying risk factors associated with frailty status. While the original study investigated the association between significant adverse outcomes and a 1-month period, this study aimed to assess the associations between the FRAIL scores and any significant adverse outcomes. It is important to note that the month of Ramadan, characterized by fasting and other unique challenges, represents an additional layer in examining frailty. In addition to changes in diet content and timing, there are changes in physical activity and exercise, which may affect their frailty conditions and outcomes (7, 8). It is hypothesized that fasting during Ramadan may contribute to variations in patient outcomes and, thus, warrants comprehensive investigation. This comprehensive perspective acknowledges the need for international studies in frailty research, given the diverse global aging populations, and underscores the relevance of frailty assessment in the unique context of Ramadan fasting.

## Methods

Ambulatory Healthcare Services (AHS) in the Abu Dhabi Emirate has structured chronic disease clinics. Within these clinics, chronic disease patients are counseled prior to Ramadan to adjust their care plan in preparation for fasting if appropriate medically. Integrating Ramadan fasting-related counseling into chronic disease patient visits is preceded by an educational event targeting physicians on new updates in this area. The IDF-DAR practical guidelines were included in this educational event, including the IDF-DAR risk stratification tool published in 2021. The AHS team requested to use the tool within the EMR system, which was built internally within CERNER EMR. The training was conducted, and physicians were encouraged to use it to help stratify diabetic patients and guide their counseling on decisions regarding fasting.

### Study design and data collection

This is a prospective observational study. Assessments were performed for all participants at two time points: within 6 weeks before Ramadan 1,444 (CE 2022) in the AHS healthcare center and again after Ramadan. Patients were included if the attending physicians used the IDF-DAR risk stratification tool as a pre-Ramadan assessment. In addition to the extracted data, a tele-interview was conducted before the start of Ramadan to determine the FRAIL score. Frailty assessment was performed using the FRAIL tool for patients who were 60 years or older. The FRAIL tool is used as a part of routine clinical care at all AHS centers and is validated with five questions that have strong evidence for predicting clinical outcomes (9–12). These questions assess fatigue, resistance (e.g., climbing stairs), ambulation (e.g., walking a few blocks), the number of chronic illnesses, and weight loss >5%. Frailty status is categorized as per the FRAIL scale score of 0–5 (1 point for each component; 0 = best, 5 = worst) into three categories: frail (3–5), pre-frail (1, 2), and robust (0).

In addition, physicians were to enter their subjective judgments about whether the patients were frail within the IDF-DAR risk stratification tool. After Ramadan, assessments were conducted via another tele-interview and a review of electronic medical records (EMRs). Data were extracted from the EMR for all patients whose risk had been assessed using the EMR form. Additional data were collected by family medicine residents through tele-interviews conducted after Ramadan.

### Sample size

Patients with diabetes who fasted all days of Ramadan or attempted to fast were included in the study. The sample size was calculated to be 166 patients, using a two-sided significance level of 95%, a power of 80%, and an effect size of 20. A total sample of 204 patients with diabetes aged 60 years old or older was included in the analysis of this study, after excluding 11 patients who were assessed before Ramadan, 10 patients who never fasted a single day, and one patient with type 1 diabetes.

### Outcome

Data were collected on fasting and medical history during Ramadan through interviews and from the EMR. In addition to demographics and clinical data, fasting status, significant health events, and time of admission into care were collected after Ramadan. Events included unplanned admissions, a history of hypoglycemia, and significant symptoms that required breaking fasting, such as dizziness, fainting, and fever. Only events occurring during Ramadan were considered in this study. The surveillance started on the 1st day of Ramadan and continued until the end of the holy month.

### Data analysis

Data analysis was performed using SPSS v27.

## Results

Of the 204 patients aged 60 years or older included in the study, 109 (53.4%), were classified as either frail or pre-frail according to the FRAIL assessment tool. In total, 20 patients (9.8%) were identified as frail, that is, 1 in 10 patients, while 89 patients (43.6%) were classified as pre-frail. The remaining 95 (46.6%) patients were robust. Overall, 37.3% of the patients were men, 62.7% were women, and 30.9% were over 70 years old. Patients were mainly UAE nationals (81.4%). In all, four patients (2%) had a history of stroke, 25 had ischemic heart disease (12.4%), and 152 (76.4%) had hypertension. Table 1 stratifies the subjects by frailty status as per the FRAIL assessment tool. The robust patients were younger, with 76.8% being 70 years or younger, compared to 25% in the same age group in the frail group.

**Table 1:** Study subjects’ characteristics.

		Robust		Prefrail		Frail		*p*-value
Age		60–70	>70	60–70	>70	61–70	>70	
	*N* (%)	73 (76.8)	22 (23.2)	63 (70.8)	26 (29.2)	5 (25)	15 (75)	<0.001
Gender		Female	Male	Female	Male	Female	Male	
	*N* (%)	57 (60)	38 (40)	56 (62.9)	33 (37.1)	15 (75)	5 (25)	0.45
Nationality		Non UAE	UAE	Non UAE	UAE	Non UAE	UAE	
	*N* (%)	20 (21.1)	75 (78.9)	13 (14.6)	76 (85.4)	5 (25)	15 (75)	0.4
IHD		No IHD	IHD	No IHD	IHD	No IHD	IHD	
	*N* (%)	89 (93.7)	6 (6.3)	76 (85.4)	11 (12.4)	11 (55)	8 (40)	<0.001
Stroke		No stroke	Stroke	No stroke	Stroke	No stroke	Stroke	
	*N* (%)	95 (100)	0	84 (96.5)	3 (3.4)	18 (94.7)	1 (5.2)	0.14
Hypertension		No hypertension	Hypertension	No hypertension	Hypertension	No hypertension	Hypertension	
	*N* (%)	24 (25.3)	69 (72.6)	22 (24.7)	64 (71.9)	1 (5)	19 (95)	0.1
CKD		No CKD	CKD	No CKD	CKD	No CKD	CKD	
	*N* (%)	87 (91.6)	8 (8.4)	72 (80.9)	17 (19.1)	12 (60)	8 (40)	0.001
On inslin		Robust		Prefrail		Frail		
	*N* (%)	Not on insulin	On insulin	Not on insulin	On insulin	Not on insulin	On insulin	
	*N* (%)	1 (1.1)	94 (98.9)	2 (2.2)	87 (97.8)	0	20 (100)	0.67
On STATIN		Robust		Prefrail		Frail		
		Not on Statin	On STATIN	Not on Statin	On STATIN	Not on Statin	On STATIN	
	*N* (%)	8 (8.4)	87 (91.6)	5 (5.6)	84 (94.4)	1 (5)	19 (95)	0.7
Duration of diabetes in years		Robust		Prefrail		Frail		
		A duration of <10	A duration of ≥10	A duration of <10	A duration of ≥10	A duration of <10	A duration of ≥10	
	*N* (%)	25 (26.3)	70 (73.7)	25 (28.1)	64 (71.9)	4 (20)	16 (80)	0.7

		Robust			Prefrail				Frail			*P*-value
Diabetes control		<7.5	7.5–9	>9	<7.5	7.5–9	>9		<7.5	7.5–9	>9	
	*N* (%)	41 (43.2)	26 (27.4)	14 (14.7)	43 (48.3)	28 (31.5)	13 (14.6)		10 (50)	5 (25)	4 (20)	0.95
IDF risk assessment category		Robust			Prefrail				Frail			
		Low risk	Moderate risk	High risk	Low risk	Moderate risk	High risk		low risk	Moderate risk	High risk	
	*N* (%)	47 (49.5)	29 (30.5)	19 (20)	37 (41.6)	33 (37.1)	19 (21.3)		4 (20)	2 (10)	14 (70)	<0.001

Although 60% of the robust group were women compared to 75% of the frail group, gender and most of the different variables in Table 1 were not found to be significant associations of frailty after adjustment for age using linear regression. Only chronic kidney diseases (CKDs), age, and history of stroke were associated with frailty (beta = 0.27, *p* = 0.011; beta = 0.041, *p* = 0.013; and beta = 1.22, *p* = 0.031, respectively) (Table 2).

**Table 2:** Significant associations with frailty.

	B	Std. error	Beta	*P*-value
CKD	0.717	0.277	0.262	0.011
AGE	0.041	0.016	0.25	0.013
Stroke	1.22	0.557	0.203	0.031

Among the total sample of patients, 22 had a significant event after the assessment. Appendix 1 lists these events as COVID-19 infection or pneumonia (27.3%), hypoglycemia (27.3%), and hyperglycemia (9.1%), which constitute the most common causes. One-third of the frail patients experienced a significant health event, including hospital admissions. The incidence in pre-frail patients was only 11.2%, and among robust patients, the incidence was 6.3%. By contrast, multiple weekly hypoglycemia occurred in 3.4% of the pre-frail patients, 1.1% among the robust patients, and none among the frail patients.

A higher frailty score was identified as the major independent risk factor [B = 0.45, OR = 1.57 (1.03–2.39), *p* = 0.033] for adverse events during Ramadan as determined by logistic regression. Other significant predictors included: number of days not fasted [B = −0.084, OR = 0.92 (0.86–0.99), *p* = 0.017] and being in the low-risk category of the DAR risk assessment tool [B = −1.35, OR = 0.26 (0.07–0.91), *p* = 0.042] (Table 3). The logistic regression model showed a good prediction for adverse outcomes, with an area under the curve (AUC) of 0.756 (0.643–0.87), a sensitivity of 59.1%, and a specificity of 82.4%.

**Table 3:** Associations of the occurrence of significant adverse events with studied factors and assessment tools.

	B	*P*-value	OR	95% CI	
Days not fasted	−0.084	0.017	0.919	0.858	0.985
High_RIsk	−0.385	0.522	0.68	0.209	2.212
	Moderate risk as reference				
Low_RIsk	−1.357	0.035	0.257	0.073	0.912
Frailty score	0.454	0.033	1.575	1.038	2.39

**Figure 1:**
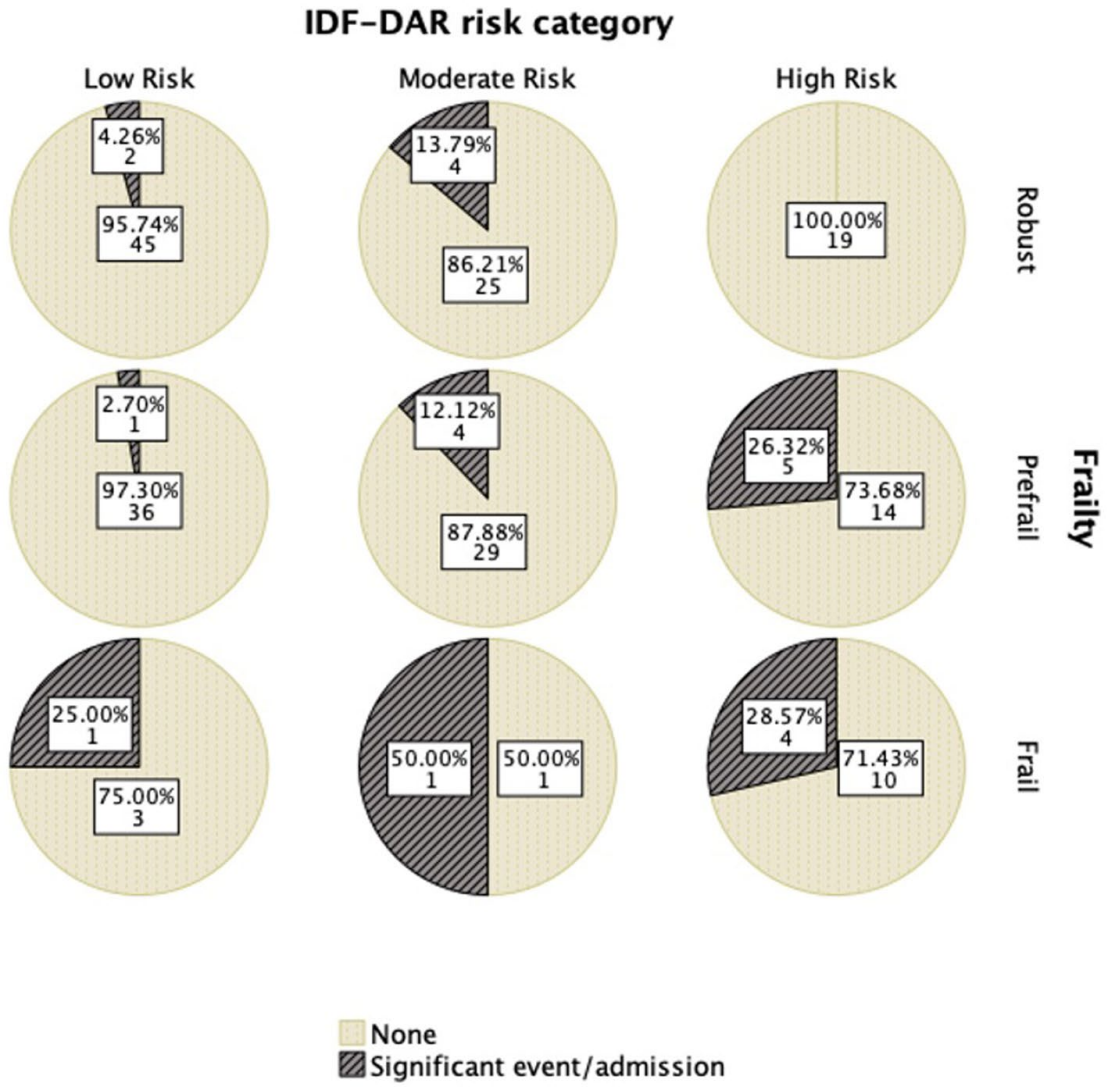
IDF_DAR category in relation to FRAIL categories.

**Figure 2:**
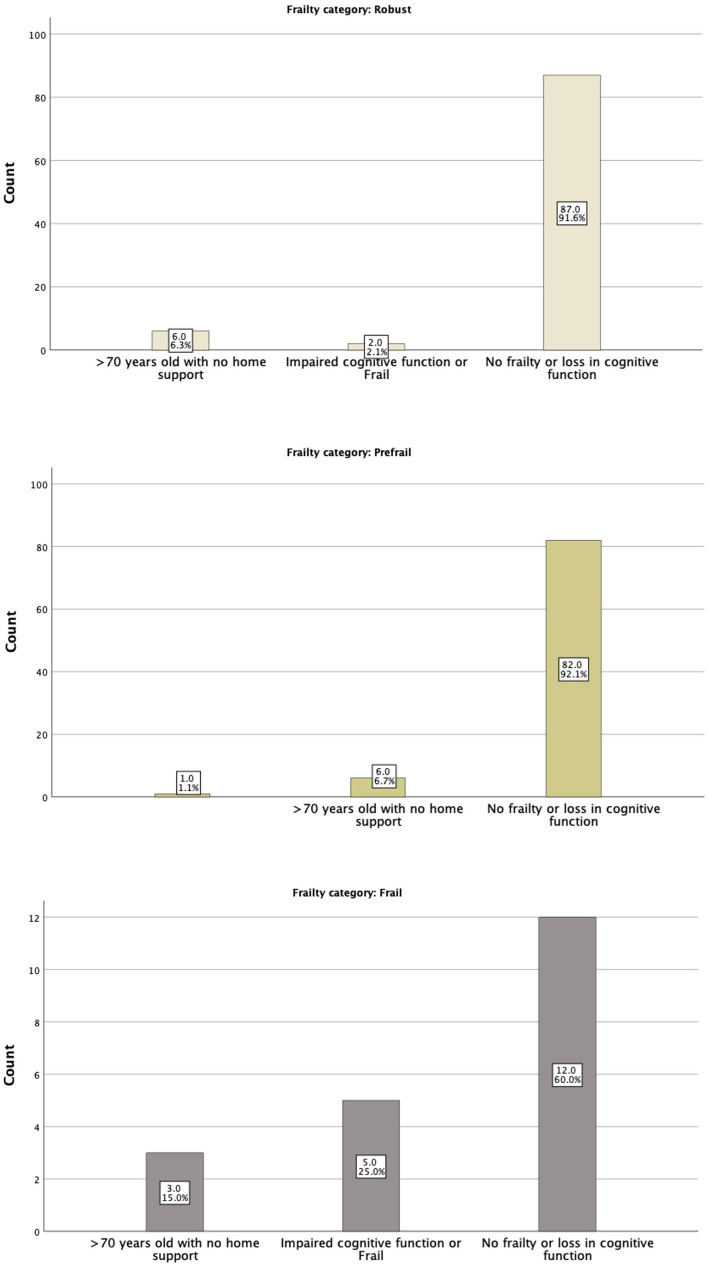
The FRAIL tool categories in relation to Physicians’ assessment.

Figure 1 shows this significant relation with the frail group having a higher DAR risk score. Robust patients had no significant event occurring, even among those in the higher-risk category. There was a clear association between frailty and DAR risk, as well as with outcomes, with pre-frail and frail patients having higher-risk categories than robust patients. In addition, DAR low-risk patients, when they were frail, had a higher likelihood of having significant events (Figure 2; Table 5).

The IDF-DAR risk assessment included two questions regarding frailty: whether the patient is over 70 years old and lacks home support available and whether the patient has impaired cognitive function or is classified as frail. Interestingly, physicians’ global assessment of a patient being frail did not match the structured FRAIL scoring well. Only 5 (25%) of the 20 patients identified as frail by the FRAIL assessment tool were judged as frail or having cognitive function impairment by physicians’ global assessment tool. Nevertheless, five of the seven identified patients by the physicians as frail or having cognitive function impairment (71.4%) were found to be in the frail category through the FRAIL assessment tool. As shown in Table 4, 6.6% of the robust patients, by physicians’ judgment, were frail, and 45.3% were pre-frail. Both the structured FRAI tool and physicians agreed that 48.1% of the patients were robust (Figure 2). It is worth noting that in logistic regression, only age [OR = 1.16 (1.01–1.23)] per year was significantly associated with the physicians’ global assessment of frailty. Comparing the sensitivity and specificity of the subjective evaluation of frailty by physicians to the frailty score, the area under the curve for each method was analyzed: the FRAIL score demonstrated better performance than the physician’s judgment with an AUC of 0.671 (0.541–0.800), a sensitivity of 57.1%, and a specificity of 74.7%, compared to an AUC of 0.513 (0.383–0.643), a sensitivity of 48%, and a specificity of 97.6%. Using the model developed from the logistic regression that included identified significant determinants, such as the FRAIL score, the AUC improved to 0.756 (0.643–0.870), with a sensitivity of 59.1% and a specificity of 82.4%.

**Table 4:** Frailty categories as per the FRAIL tool in relation to Physicians’ assessment.

		Physicians assessment			Total
FRAIL tool categories		No frailty or loss in cognitive function	>70 years old with no home support	Impaired cognitive function or frail	
Robust	Count	87	6	2	95
	% within frail cat	91.60%	6.30%	2.10%	100.00%
	% within calculator frail	48.10%	40.00%	28.60%	46.80%
	% of total	42.90%	3.00%	1.00%	46.80%
Prefrail	Count	82	6	0	88
	% within frail cat	93.20%	6.80%	0.00%	100.00%
	% within calculator_frail	45.30%	40.00%	0.00%	43.30%
	% of total	40.40%	3.00%	0.00%	43.30%
Frail	Count	12	3	5	20
	% within frail cat	60.00%	15.00%	25.00%	100.00%
	% within calculator frail	6.60%	20.00%	71.40%	9.90%
	% of total	5.90%	1.50%	2.50%	9.90%
Total	Count	181	15	7	203
	% within frail cat	89.20%	7.40%	3.40%	100.00%
	% within calculator frail	100.00%	100.00%	100.00%	100.00%
	% of total	89.20%	7.40%	3.40%	100.00%

**Table 5:** Frailty assessment by IDF Dar tool.

		Low risk	Moderate risk	High risk	
Frail cat	Robust	47	29	19	95
		52.80%	45.30%	36.50%	46.30%
	Prefrail	38	33	19	90
		42.70%	51.60%	36.50%	43.90%
	Frail	4	2	14	20
		4.50%	3.10%	26.90%	9.80%

## Discussion

Frailty is a pervasive and significant concern among patients aged 60 and older with diabetes. More than half of the patients in our study were identified as frail or pre-frail, highlighting the importance of considering frailty in the management of this population. Recognizing frailty is essential as it informs the selection of appropriate interventions, including invasive procedures or drug treatments. Such knowledge is invaluable for steering the care of frail elderly individuals toward goal-directed and tailored approaches (13). The prevalence reported in this study is higher than the reported prevalence in the region and the world, from 4 to 59.1% (14, 15). In a study in Saudi Arabia, the FRAIL tool was found to be culturally adapted to older Saudi adults and demonstrated acceptable internal consistency and test–retest reliability but was not validated (16). This study relates to the outcome and, therefore, validates this tool in the Arabic diabetic population.

While age is a non-modifiable risk factor, the significant associations of frailty score with stroke and CKD highlight these two conditions as a priority in clinical care to mitigate and prevent further deterioration in quality status. Effective strategies to prevent these two conditions must also be invested in. This relationship underscores the physical impact of these conditions on patients’ overall health and the limitations they impose on nutrition and physical activities. The implications of these findings are profound, as early detection and management of these conditions have been proven to be feasible and are key in preventing frailty.

This study identified a strategy that represents a structured and validated tool for assessing frailty. The FRAIL score demonstrated better predictability of significant health outcomes compared to physicians’ global judgments. This observation aligns with a growing body of evidence (3, 5, 17) and underscores the importance of incorporating the FRAIL tool into clinical practice. This recommendation is consistent with established guidelines such as those of the British Geriatric Society, which advocates for assessing frailty in older adults (18).

While this study offers valuable insights into frailty among patients with diabetes, it also calls for further research in various patient populations to understand the impact of diabetes on frailty comprehensively. Although increased frailty was significantly associated with re-hospitalization and discharge to an institution in a study by Leken and McCoy, only diabetes was significantly associated with in-hospital mortality (19). Previous studies have demonstrated the adverse effects of pre-frailty and frailty on the risk of mortality, cardiovascular events, and healthcare utilization among patients with type 2 diabetes (17, 20). Detecting frailty and implementing appropriate interventions hold the potential to improve the prognosis and quality of life in older patients (21).

One notable feature of this study is the use of the FRAIL tool, which is a simple and concise assessment tool. It has been employed in previous research and has been found to confer a high risk of in-hospital mortality in other studies (22). Its simplicity makes it highly practical for use in primary care settings, enhancing its potential for widespread application. This study showed that the performance in predicting adverse events improved across the three methods evaluated. Physician judgment had the lowest performance among them. In contrast, the FRAIL score exhibited good performance, particularly when combined with other relevant associations identified in the study. This finding calls for more studies in developing and validating risk scores in frailty. Other factors may contribute to it in varying degrees from different geographical areas and cultures.

Moreover, this study is the first that used the FRAIL score in assessing the outcome during the potentially stressful fasting during Ramadan. This adds valuable insight into the interaction between frailty and fasting, particularly in the context of Ramadan.

However, it is essential to recognize that while tools such as the FRAIL score are valuable for assessing frailty, the variable impacts of aging that contribute to frailty are intertwined with a robust set of social determinants (2). These determinants including socio-economic factors, healthcare access, and environmental conditions play a critical role in the overall frailty picture. Future research should aim to further delineate the interplay between these determinants and frailty, providing a more comprehensive understanding of the condition.

There are a few limitations of this study that are important to note. The majority of participants are UAE nationals, which may limit the applicability of the findings to other ethnic or geographic populations. A more diverse sample would help validate the results across different demographic groups. Moreover, a larger and more diverse sample size could improve the generalizability of the findings.

The study focuses on adverse events during Ramadan, which is a relatively short period. Long-term outcomes and the impact of frailty beyond Ramadan are recommended as future areas for research that could provide a more comprehensive understanding of the risks associated with frailty in diabetic patients. Finally, while the significant disparity between the FRAIL tool and physicians’ global assessment was evident and carries important clinical implications, this inconsistency underscores a critical area for further research. Investigating the underlying causes of such variations and developing targeted interventions to address these discrepancies could enhance the accuracy and effectiveness of frailty assessments.

## Conclusion

Frailty is prevalent among elderly patients with diabetes, particularly those with comorbid conditions. Our study underscores the significance of frailty when assessing the risk of fasting during Ramadan. Notably, the disparity between subjective and objective frailty assessments emphasizes the need for standardized evaluation methods. Using the FRAIL tool is recommended for patients aged 60 or older with diabetes in Abu Dhabi.

## Data Availability

The original contributions presented in the study are included in the article/Supplementary material, further inquiries can be directed to the corresponding author.
