# Supplementary material for: Frailty assessment and outcomes in primary care for patients with diabetes during Ramadan: implications for risk evaluation and care plans
Source: Front Med (Lausanne). 2024 Sep 30;11:1426140. doi: 10.3389/fmed.2024.1426140 (PMC11475290; doi:10.3389/fmed.2024.1426140)
Supplement: Supplementary file 1 [file Data_Sheet_1.docx]

Appendix 1

| **Significant Event-mention** | **Number of events** |
| --- | --- |
| COVID infection | 6 (27.27%) |
| hypoglycemia | 6 (27.27%) |
| Hyperglycemia, one required ICU admission | 2 (9.1%) |
| Stroke | 1 (4.54%) |
| Surgical removal of failed dental implant | 1 (4.54%) |
| Total knee arthroplast | 1 (4.54%) |
| Urinary Trackt Infection, sepsis | 1 (4.54%) |
| Acute Kidney Imparment. Diahrrae with dehydration | 1 (4.54%) |
| Asthma with dehydration | 1 (4.54%) |
| Diagnosed with lumbar stenosis | 1 (4.54%) |
| Fluid overload | 1 (4.54%) |
| Total | 22 (100%) |
